# A Study on Chinese EFL Teachers' Work Engagement: The Predictability Power of Emotion Regulation and Teacher Resilience

**DOI:** 10.3389/fpsyg.2021.735969

**Published:** 2021-08-27

**Authors:** Fei Xie

**Affiliations:** School of College English Teaching and Research, Henan University, Kaifeng, China

**Keywords:** EFL teachers, work engagement, emotion regulation, resilience, teacher's perceptions

## Abstract

Employing a sequential mixed-methods design, the current study examined the role of Chinese EFL teachers' emotion regulation and resilience in predicting their work engagement. To this end, 314 Chinese EFL teachers with various academic degrees and teaching experiences were opted from different schools, institutes, and universities of China. To obtain the quantitative data, Utrecht Work Engagement Scale (UWES), Connor–Davidson Resilience Scale (CD-RISC), and Emotion Regulation Questionnaire (ERQ) were electronically distributed among participants. Performing correlational analyses, a strong association was found between teacher resilience and work engagement. The inspection of the correlations also revealed a moderate correlation between cognitive reappraisal and resilience as well as cognitive reappraisal and work engagement. To probe the predictability power of teacher resilience and emotion regulation (cognitive reappraisal), structural equation modeling (SEM) was performed. The results of the SEM analysis demonstrated that Chinese EFL teachers' work engagement was predicted significantly and favorably by their resilience. Using semi-structured interviews, some qualitative data were also collected to fully understand Chinese EFL teachers' perceptions of work engagement. The thematic analysis (TA) of Chinese EFL teachers' responses to interview questions resulted in two main themes and 14 sub-themes, revealing extrinsic and intrinsic factors contributing to teaching engagement. The findings of TA illuminated that both personal resources and job resources can predict teaching engagement. The pedagogical implications for administrators and teacher trainers are further discussed.

## Introduction

Teaching is typically considered as one of the most challenging vocations (McIntyre et al., 2017). However, the majority of teachers are incredibly devoted and passionate about their profession. Schaufeli et al. (2002) referred to this sense of enthusiasm and commitment as work engagement, referring to “a positive, fulfilling and work-related state of mind that is characterized by vigor, dedication and absorption dimensions” (p. 75). As put forward by Schaufeli et al. (2002), those who are more engaged with their profession are more energetic, committed to and enthusiastic about their work, and highly interested in what they do. In this regard, Cardwell (2011) also stated that high levels of teacher professional engagement have a favorable impact on teaching quality. Due to the pivotal role of teachers' engagement in the adequacy of their instruction, a growing body of research has been conducted on the factors contributing to EFL/ESL teachers' work engagement (e.g., Hultell and Gustavsson, 2011; Mérida-López et al., 2020; Greenier et al., 2021). To pursue this line of inquiry, the present study aims to examine the role of emotion regulation and teacher resilience as predictors of Chinese EFL teachers' work engagement.

Emotion regulation, as a potential predictor of teacher work engagement, pertains to “various cognitive, physiological, and behavioral processes that a person employs to regulate his/her emotional expressions and experiences” (Gross and John, 2003, p. 349). Teachers experience a variety of emotions in instructional-learning contexts: happiness when weak students eventually realize a complex concept, contentment when students get the correct answer, pleasure when they witness student collaboration, disappointment at students' reluctance, and frustration when students do not follow the rules and guidelines of the learning environment (Sutton and Wheatley, 2003; Oplatka, 2009). To create a friendly and productive instructional-learning atmosphere, teachers should effectively navigate these positive and negative emotions (Li and Xu, 2019; Braun et al., 2020; Dewaele and Li, 2020; Wang and Derakhshan, 2021). To put it differently, regulating emotional experiences is one of the important pillars of teacher success (Wang and Hall, 2021).

Along with emotion regulation, another factor that may lead teachers to become more engaged in their profession is resilience (Williams, 2003). Bobek (2002) characterized resilience as “the ability to adjust to varied situations and increase one's competence in the face of adverse conditions” (p. 202). Mansfield et al. (2016) also referred to resilience as a personal trait that enables teachers to navigate the difficulties and challenges of instruction and succeed rather than simply survive in their profession. As put forward by Polat and Iskender (2018), resilient teachers receive a great deal of personal pleasure and satisfaction from their vocation.

While several studies have been conducted on different factors contributing to teachers' work engagement (e.g., Hultell and Gustavsson, 2011; Bao et al., 2021; Greenier et al., 2021), two important variables of emotion regulation and teacher resilience have received less attention. Moreover, no empirical research has probed these variables simultaneously to examine the role of emotion regulation and teacher resilience in predicting teachers' work engagement. To bridge these lacunas, the current study attempted to examine the role of emotion regulation and teacher resilience as predictors of Chinese EFL teachers' work engagement.

## Literature Review

### Work Engagement

Work engagement as a motivational construct is defined as “a positive, fulfilling, work-related state of mind that is characterized by vigor, dedication, and absorption” (Schaufeli et al., 2002, p. 75). Vigor, as the first dimension of work engagement, pertains to “high levels of energy and mental resilience while working, the willingness to invest effort in one's work, and persistence also in the face of difficulties” (Hakanen et al., 2006, p. 498). The second dimension of work engagement is called dedication, which is described as a sense of “significance, enthusiasm, inspiration, pride, and challenge” (Hakanen et al., 2006, p. 499). Finally, the third key dimension of work engagement, absorption, is characterized by “being fully concentrated and happily engrossed in one's work, whereby time passes quickly and one has difficulties with detaching oneself from work” (Hakanen et al., 2006, p. 499). Unlike burnout that has detrimental effects on individuals' work performance, work engagement as a positive aspect of work-life favorably affects individuals and institutions (González-Romá et al., 2006).

Following the recent positive psychology movement (MacIntyre et al., 2016; Dewaele et al., 2019; Wang et al., 2021), many scholars start working on different positive aspects of work-related health outcomes, notably work engagement (e.g., Hultell and Gustavsson, 2011; Runhaar et al., 2013; Mérida-López and Extremera, 2017; Burić and Macuka, 2018; Greenier et al., 2021). Hultell and Gustavsson (2011), for instance, investigated factors influencing pre-service teachers' work engagement. Using surveys, the required data was gathered from 1,290 Swedish teachers. To analyze the obtained data, the researchers performed a series of multiple regression analyses. The results of the analysis delineated that job resources were closely associated with teachers' work engagement. In another study, Minghui et al. (2018) studied teacher work engagement in relation to teacher efficacy and social support. To do so, 1,027 Chinese teachers were invited to respond to three reliable questionnaires of the constructs. Based on the results of correlation analysis, the researchers found that there was an interrelationship between teacher efficacy, social support, and teacher work engagement. In a similar vein, Van Der Want et al. (2019) examined the probable association between teachers' identity, self-efficacy, and work engagement. To this aim, 29 teachers were selected from different schools of Netherlands. The researchers employed some questionnaires and semi-structured interviews to gather participants' viewpoints regarding the association between identity, self-efficacy, and work engagement. Analyzing participants' responses, they reported that teachers' identity and self-efficacy can significantly and positively predict their work engagement.

In a recent cross-cultural study, Greenier et al. (2021) also attempted to investigate the role of psychological well-being and emotion regulation as antecedents of Iranian and British language teachers' work engagement. In doing so, there pre-developed questionnaires were distributed among 363 Iranian and British English language teachers. In order to triangulate data, 11 Iranian (*n* = 6) and British language teachers (*n* = 5) were also interviewed. Analyzing participants' responses, the researchers found that both psychological well-being and emotion regulation can contribute to increased English language teacher engagement.

### Teacher Resilience

The concept of resilience is generally characterized as “using energy productively to achieve educational goals in the face of adverse conditions” (Patterson et al., 2004, p. 4). More specifically, teacher resilience is conceptualized as teachers' capacity to adapt to diverse environments and enhance their competence in the face of difficulties (Bobek, 2002). Mansfield et al. (2016) also referred to teacher resilience as a personal characteristic that assists teachers in managing the difficulties and challenges of instruction. In a more comprehensive definition, Beltman (2015) conceptualized teacher resilience in terms of *capacity, process*, and *outcome*. Capacity pertains to the teacher's ability to employ existing facilities to overcome difficulties and obstacles. The process deals with the situation in which teachers' individual qualities interact with environmental factors to use certain techniques in the face of difficulties. Finally, the outcome relates to the ultimate performance of a resilient teacher as a teacher with more dedication, satisfaction, academic improvement, and well-being (Beltman, 2015, p. 25).

In an attempt to characterize resilient teachers, Howard and Johnson (2004) noted that resilient teachers are those who continually exhibit “a sense of agency, moral purpose, a strong support group, and a sense of accomplishment” (p. 12). In another attempt, Stanford (2001) introduced *high morale* as one of the key characteristics of resilient teachers. To him, resilient teachers are those who have positive attitudes toward their profession. Bobek (2002) also identified *sense of humor* as another distinguishing feature of resilient teachers. He noted that a teacher who fosters a sense of humor and the capacity to laugh at their own mistakes provides a great outlet for frustrations. In this regard, Day (2008) proposed that resilient teachers are those who have the necessary skills to succeed in challenging conditions, are effective at classroom management, and build positive relationships with their pupils.

Given the fact that teacher resilience can significantly contribute to favorable educational outcomes (Day and Gu, 2014), some researchers, notably those interested in education, have conducted some academic investigations on this variable (e.g., Beltman, 2015; Vance et al., 2015; Peixoto et al., 2018; Parsi, 2019; Razmjoo and Ayoobiyan, 2019; Fathi and Saeedian, 2020; Zhang et al., 2020). Parsi (2019), for instance, probed the association between Iranian EFL teachers' resilience and their creativity. In doing so, 120 Iranian EFL teachers voluntarily took part in this study. To collect the required data, the “Creativity Fostering Teacher Index” and the “Teacher Resilience Scale” were distributed among participants. Pearson product-moment correlation was performed to analyze the gathered data. The results of data analysis demonstrated that there was a favorable association between EFL teachers' creativity and resilience. Moreover, regression analyses were also performed to examine the role of teacher resilience as the antecedent of creativity. The outcomes of regression analyses indicated that teachers' resilience can considerably and favorably predict their creativity. In another study, Razmjoo and Ayoobiyan (2019) studied EFL teachers' resilience in relation to their self-efficacy. To do this, 92 EFL teachers were selected from a private language institute. To gather data, participants were asked to fill out two validated instruments of “Teacher Resilience” (Tschannen-Moran and Hoy, 2001) and “Self-efficacy” (Connor and Davidson, 2003). To analyze participants' responses to the aforementioned scales, Pearson product-moment correlations were performed. Based on the results of analyses, the researchers found that self-efficacy can positively influence language teachers' resilience. In a similar vein, Fathi and Saeedian (2020) explored the impact of resilience and self-efficacy in EFL teachers' burnout. To this aim, 213 Iranian EFL teachers with different teaching experiences were asked to complete three pre-developed questionnaires. To examine the associations among the questionnaires correlational analyses were conducted. The results of analyses revealed a significant interrelationship between teachers' sense of self-efficacy, resilience, and burnout. The results of correlational analyses were also confirmed by Structural Equation Modeling (SEM) results.

### Emotion Regulation

The definition of emotion regulation appears to be a source of debate in the literature. That is, various scholars have conceptualized emotion regulation in different ways. For instance, Cole et al. (1994) characterized emotion regulation as “the ability to respond to the ongoing demands of experience with the range of emotions in a manner that is socially tolerable and sufficiently flexible to permit spontaneous reaction as well as the ability to delay spontaneous reactions as needed” (p. 75). However, Thompson et al. (2008) referred to this concept as the internal and external mechanisms through which people can modify, assess, or manage their emotions to attain their goals.

In characterizing the concept of emotion regulation, several models were proposed. Among them, one can refer to *Hot/Cool System Model* (Metcalfe and Mischel, 1999) as the most comprehensive model of emotion regulation. According to this model, the concept of emotion regulation encompasses a hot and a cool system. The cool system is “cognitive, complex, slow, contemplative, and emotionally neutral” (Sutton and Harper, 2009, p. 391). It is composed of a series of informational *cool nodes* that are intrinsically intertwined with one other. These cool nodes generate *rational, reflective*, and *strategic behaviors*. On the other hand, the hot system, which is made of some *hot spots*, is a *go* or *hot button* system that paves the way for faster emotion processing (Sutton and Harper, 2009). This model suggests that those teachers who effectively control their emotions alter the *hot representation* of the immediate circumstance to a cool one through disregarding the stimulus or interpreting it in a different way (Sutton, 2004).

As put forward by Gross (1998), teachers generally use emotion-regulation strategies to establish positive relationships with their pupils and to portray themselves in the best possible light. In this regard, Sutton (2004) also classified emotion regulation strategies into two main categories, including *Antecedent-Focused Strategies* and *Response-Focused Strategies*. Antecedent-focused strategies are “commonly used by teachers before the initiation of the emotional arousal stages through cognitive change, situation selection, situation manipulation, and attention deployment.” In contrast, response-focused strategies are “typically employed by teachers after the initiation of the emotional arousal stages” (Greenier et al., 2021, p. 10). Among different strategies teachers employ in instructional-learning contexts in order to navigate positive and negative emotions, one can refer to *cognitive reappraisal* and *expressive suppression* as two major instances of emotion-regulation strategies (Gross and John, 1998, 2003). Cognitive reappraisal is characterized as the endeavor “to conceptualize an emotion-eliciting condition in a manner that changes its meaning and emotional impact” (Cutuli, 2014, p. 175). Expressive suppression refers to any attempt with the aim of concealing, inhibiting, or decreasing emotion-expressive behavior (Gross and John, 2003).

As previously mentioned the concepts of resilience and emotion regulation received limited attention in the realm of L2 research. However, compared to teacher resilience, emotion regulation has been the focus of more empirical research (e.g., Brackett et al., 2010; Jiang et al., 2016; Akbari et al., 2017; Fathi and Derakhshan, 2019; Katana et al., 2019; Braun et al., 2020; Chang, 2020; Han et al., 2020; Fathi et al., 2021). Brackett et al. (2010), for instance, investigated the associations among teachers' emotion-regulation ability, job satisfaction, and burnout. To do so, 123 British school teachers voluntarily participated in this study. Three questionnaires of emotion-regulation ability, job satisfaction, and burnout were distributed among participants to gather the required data. Analyzing teachers' responses to the aforementioned scales, the researchers reported that emotion regulation ability was correlated significantly and positively with job satisfaction and one dimension of burnout.

By the same token, Fathi and Derakhshan (2019) have attempted to probe into the role of emotional regulation and teacher self-efficacy as antecedents of teaching stress. In doing so, 256 EFL teachers were selected from different Iranian universities, schools, and institutes. To obtain data, participants voluntarily completed the valid instruments of the three variables. The analysis of participants' responses to the aforementioned scales indicated that there was a considerable association between teachers' self-efficacy, emotional regulation, and their teaching stress. The results of analyses also showed that both self-efficacy and emotional regulation were strong predictors of teaching stress.

In a similar vein, Han et al. (2020) examined the relationships between teachers' emotional regulation strategies (i.e., cognitive reappraisal & expressive suppression), well-being, and their job characteristics. To this end, 643 university teachers were invited to complete some validated scales. Performing correlational analyses, the relationships between the variables were examined. The findings of analyses demonstrated that teaching support and emotional job demands positively influenced their well-being. The results also revealed that teachers' cognitive reappraisal was useful to their well-being.

The current empirical study is warranted due to a number of research gaps in the existing literature. First and foremost, despite the fact that several studies have been dedicated to teacher work engagement and its antecedents, a limited number of studies have been conducted on EFL/ESL teachers' academic engagement, notably Chinese language teachers. Second, the concept of resilience, as a probable antecedent of teacher work engagement, appears to be in its infancy in the field of educational research. Finally, no empirical study has been carried out to explore the associations among teacher resilience, emotion regulation, and work engagement. To fill these gaps, the present study aims at examining the impact of emotion regulation and teacher resilience in Chinese EFL teachers' work engagement.

## Research Questions

1. Are there any significant associations among emotion regulation, resilience, and Chinese EFL teachers' work engagement?
2. To what extent can Chinese EFL teachers' work engagement be predicted by their resilience and emotion regulation?

## Method

### Participants

To collect the required data, a total number of 314 Chinese EFL teachers with various academic degrees (i.e., 75% Master of Arts, 16% Bachelor of Arts, 7% Ph.D., 1% Associate of Arts, 1% Diploma) and different teaching experiences (*M* = 17) voluntarily took part in this study. To enhance the generalizability of the outcomes, the participants were randomly selected from both genders (82% females, 18% males), different majors (i.e., Applied Linguistics, Linguistics, English Language Literature, English Language Translation, TESOL, TEFL, Majors Other than English), different age levels (*M* = 42), and different provinces of China.

The aim of the study and the method of data collection were explained to participants. To gather the consent of participants, the consent forms were sent to them through WeChat (a Chinese multi-purpose messaging app). The respondents were assured that their information would be kept confidential and be used solely in the current inquiry. They were also asked if they were willing to take part in follow-up interviews.

### Instruments

#### Utrecht Work Engagement Scale (UWES)

Chinese EFL teachers' work engagement was measured via Schaufeli et al. (2002) Utrecht Work Engagement Scale (UWES). This scale encompasses 17 items to which participants respond on a 7-point Likert scale ranging from 0 (*never*) to 6 (*always*). The UWES includes three subscales, namely “*Vigor (VI)*” (items 1–6), “*Dedication” (DE)* (items 7–11), and “*Absorption (AB)”* (items 12–17). The composite reliability of UWES was estimated to be 0.95 in the current study.

#### Connor–Davidson Resilience Scale (CD-RISC)

The 10-item version of Connor–Davidson Resilience Scale (CD-RISC) validated by Campbell-Sills and Stein (2007) was employed to measure Chinese EFL teachers' resilience. The respondents' answers to the items can vary on a 5-point Likert-type scale, from 0 (Not true at all) to 4 (True nearly all the time). The composite reliability of CD-RISC in this study was 0.87.

#### Emotion Regulation Questionnaire (ERQ)

To assess Chinese EFL teachers' emotion regulation, Emotion Regulation Questionnaire (ERQ) designed by Gross and John (2003) was utilized. The ERQ comprises 10 items, designed to determine participants' inclination to manage their emotions in two distinct aspects, namely “*Expressive Suppression*” (four items), and “*Cognitive Reappraisal*” (six items). The questionnaire uses a 7-point Likert scale, varying in responses from 1 (Strongly Disagree) to 7 (Strongly Agree).

#### Semi-structured Interviews

To achieve triangulation and gain a deeper understanding of Chinese EFL teachers' viewpoints toward teaching engagement, some semi-structured interviews were performed. An interview guide with three predefined and some follow-up questions were designed to conduct such interviews. Prior to interviewing the participants, some interview sessions were conducted with non-participants to identify the drawbacks of the interview guide. Based on the results of the pilot sessions, the researcher removed the identified deficiencies and finalized the interview guide.

### Data Collection Procedure

Employing a sequential mixed-methods design, the two research questions were addressed. At the first stage, the quantitative data were obtained via the electronic version of the questionnaires (UWES, CD-RISC, ERQ) distributed through WeChat among 314 Chinese EFL teachers. Prior to completing the questionnaires, the participants were asked to fill out the consent forms sent to them electronically.

At the second stage, to gather the qualitative data, semi-structured interviews were held with 12 participants, who were inclined to take part in interview sessions. As all interviewees had a good command of English, the interview sessions were held in English. Due to the COVID-19 pandemic, all interview sessions were performed in a virtual platform to follow the health protocols and guidelines. For further thematic analysis, the interview sessions were recorded and fully transcribed.

### Data Analysis

#### Quantitative Analysis

Prior to commencing the analysis, the gathered data went through some pre-processes to exclude the problematic data. Then, to make sure of the construct validity, CFA was performed. Composite reliability was also employed to estimate the reliability of scales. Next, to examine the associations among Chinese EFL teachers' emotion regulation, resilience, and work engagement, the Pearson correlation procedure was run. Finally, to probe the role of Chinese EFL teachers' emotion regulation and resilience as predictors of their work engagement, structural equation modeling (SEM) was performed, through the *MPLUS software* (version 8).

#### Qualitative Analysis

To analyze the participants' answers to interview questions, *a thematic analysis* (TA) was employed. For the sake of credibility (Friedman, 2012), all phases of TA were carried out by two applied linguists (i.e., the authors of the present study) who have undertaken extensive research in this area. Given the fact that employing a “*Computer-Assisted Qualitative Data Analysis Software*” (CAQDAS) can also remarkably enhance the credibility of the coding process (Baralt, 2012), the analysts employed *MAXQDA software* (version 2020) to codify the interviewees' responses.

Prior to initiating the coding process, the interviewees were numbered, and their responses were compiled in a single word file. In the first phase, known as open coding, each analyst individually read the transcribed data and generated some initial codes, accordingly. Then, in the phase of axial coding, they compared the initial codes and grouped them under some related themes. Finally, in the phase of selective coding, the analysts classified the generated themes by putting them under higher-order headings. The thematic analysis of participants' perceptions toward teaching engagement culminated in two main themes (i.e., Intrinsic factors, Extrinsic factors). Since all stages of codification were performed by the researcher and an applied linguist, the level of agreement between them was estimated. Utilizing *Krippendorff's alpha (*α*)*, an inter-coder agreement coefficient of 0.97 was obtained, suggesting that coders had attained a high level of agreement.

## Results

### The Quantitative Results

Prior to initiating the analysis, some pre-processes were conducted to detect the problematic data. Initially, 314 solid answers were received from the administration of the questionnaires. There was no missing response in the data, and the data was initially checked for patterns. Consequently, 16 cases with constant pattern (Case No. 4, 17, 49, 97, 98, 147, 165, 180, 193, 206, 207, 240, 276, 277, 283, and 309), four cases with decreasing pattern (Case No. 61, 211, 241, and 287), and three cases with increasing pattern (Case No. 2, 65, and 298) were detected and excluded. Then, the standard deviation (SD) of respondents' answers was calculated and those with values below 0.5 were examined for disengagement. Fortunately, no such a case was found. As a result of data screening, 291 respondents were remained for the main analysis.

First, CFA was conducted to make sure of the construct validity. The initial model had two construct (ERQ, UWES) with items in second order and one (CD-RISC) with first order. However, the evaluation of the validity revealed serious problems for ERQ in terms of both convergent (low composite validity and AVE) and divergent validity (high correlation with CD-RISC). Therefore, the components of ERQ were put into the model separately. Then, each construct was examined for non-significant loadings in unstandardized estimation and/or low estimates (below 0.5) in standardized estimation. As Table 1 demonstrates, no non-significant unstandardized estimates were found. However, four items with low standardized estimates (i.e., item 1 in cognitive reappraisal, item 2 in expressive suppression, item 5 in resilience scale, and item 1 in dedication) were excluded before going forward.

**Table 1:** Unstandardized and standardized estimates of the initial CFA model.

			**Unstandardized**				**Standardized**
			**Estimate**	**S.E**.	**C.R**.	***P***	**Estimate**
VI	< –	UWES	1.000				0.956
DE	< –	UWES	0.428	0.073	5.883	0.000	0.918
AB	< –	UWES	0.940	0.085	11.123	0.000	0.897
ER1	< –	CR	1.000				0.487
ER3	< –	CR	1.009	0.141	7.132	0.000	0.598
ER5	< –	CR	1.136	0.154	7.401	0.000	0.639
ER7	< –	CR	1.213	0.155	7.836	0.000	0.716
ER8	< –	CR	1.293	0.158	8.157	0.000	0.787
ER10	< –	CR	1.280	0.158	8.099	0.000	0.773
ER2	< –	ES	1.000				0.455
ER4	< –	ES	1.172	0.210	5.560	0.000	0.500
ER6	< –	ES	1.896	0.296	6.414	0.000	0.793
ER9	< –	ES	1.698	0.265	6.414	0.000	0.694
RS1	< –	CD.RISC	1.000				0.636
RS2	< –	CD.RISC	1.247	0.124	10.046	0.000	0.696
RS3	< –	CD.RISC	1.039	0.122	8.541	0.000	0.572
RS4	< –	CD.RISC	0.843	0.109	7.740	0.000	0.510
RS5	< –	CD.RISC	0.883	0.117	7.516	0.000	0.493
RS6	< –	CD.RISC	0.980	0.103	9.493	0.000	0.649
RS7	< –	CD.RISC	1.273	0.125	10.175	0.000	0.707
RS8	< –	CD.RISC	1.448	0.134	10.816	0.000	0.766
RS9	< –	CD.RISC	1.393	0.131	10.615	0.000	0.747
RS10	< –	CD.RISC	1.287	0.121	10.680	0.000	0.753
VI1	< –	VI	1.000				0.714
VI2	< –	VI	1.064	0.082	13.046	0.000	0.792
VI3	< –	VI	0.792	0.066	12.069	0.000	0.733
VI4	< –	VI	0.945	0.080	11.770	0.000	0.715
VI6	< –	VI	1.156	0.078	14.816	0.000	0.903
DE1	< –	DE	1.000				0.367
DE2	< –	DE	2.336	0.370	6.307	0.000	0.847
DE3	< –	DE	2.527	0.397	6.363	0.000	0.887
DE4	< –	DE	2.394	0.381	6.277	0.000	0.828
DE5	< –	DE	2.369	0.374	6.338	0.000	0.868
AB1	< –	AB	1.000				0.771
AB2	< –	AB	0.919	0.067	13.655	0.000	0.773
AB3	< –	AB	0.738	0.084	8.768	0.000	0.520
AB4	< –	AB	0.881	0.073	12.028	0.000	0.692
AB5	< –	AB	1.028	0.069	14.801	0.000	0.828
AB6	< –	AB	0.899	0.080	11.254	0.000	0.653

**Figure 1:**
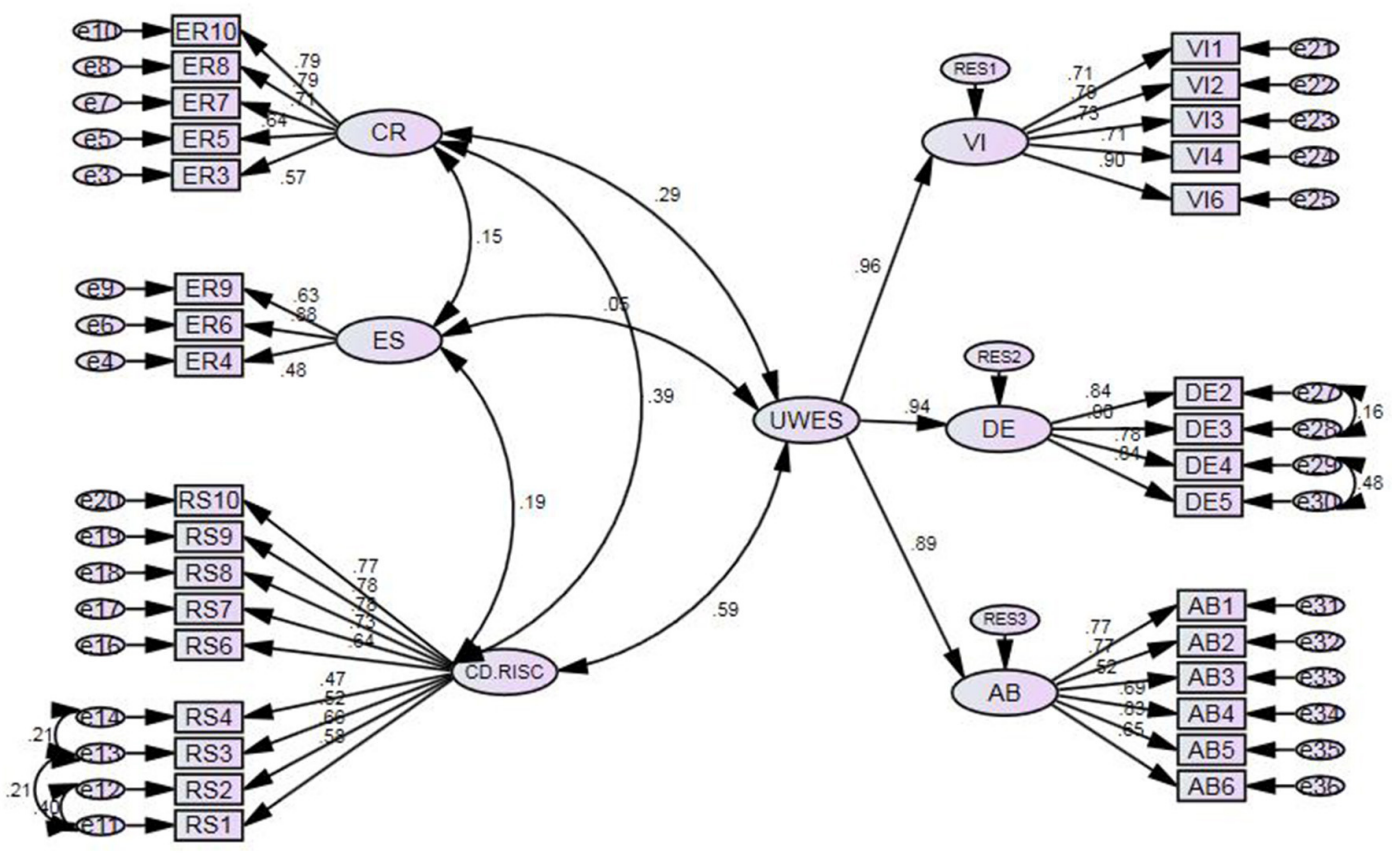
The final modified CFA model with standardized estimates.

Second, the modification indices with the threshold of 10 were examined and the suggestions that were not opposed to the literature were applied. Figure 1 portrays the final modified CFA model.

Third, the composite reliability (CR) and discriminant validity for each factor was examined. As Table 2 illustrates, all of the variables had CR values above 0.7, revealing high degree of reliability. Moreover, the square root of average variance extracted (AVE) (the bold values in the table) was above inter-correlations of the factors.

**Table 2:** Composite reliability and discriminant validity of the factors.

**Factors**	**Composite Reliability**	**Fornell-Larcker Criterion**			
		**CR**	**ES**	**CD-RISC**	**UWES**
CR	0.830	**0.706**			
ES	0.713	0.149^*^	**0.684**		
CD-RISC	0.876	0.387^**^	0.190^*^	**0.669**	
UWES	0.950	0.288^**^	0.049	0.585^**^	**0.930**

* *Correlation is significant at p < 0.05*.

** *Correlation is significant at p < 0.01. Moreover, the square root of average variance extracted (AVE) (the bold values) was above inter-correlations of the factors*.

The inspection of the correlations documented that there are significant correlations between all pairs of factors except for expressive suppression (ES) and work engagement (UWES). As shown in Table 2, there were strong correlations between CD-RISC and UWES (r = 0.585). Furthermore, it is portrayed that there were moderate correlations between cognitive reappraisal and resilience (r = 0.387) as well as cognitive reappraisal and work engagement (r = 0.288). Moreover, significant, but weaker, correlations were found between cognitive reappraisal and expressive suppression (r = 0.149) as well as expressive suppression and resilience (r = 0.190). The only non-significant correlation was between expressive suppression and work engagement (r = 0.049). Since the relationship between expressive suppression and work engagement was not significant (Table 2), the construct of expression suppression was excluded from further analyses.

Finally, to examine the role of cognitive reappraisal and resilience as antecedents of Chinese EFL teachers' work engagement, SEM was employed. Various fit indices, including CMIN/df, RMSEA, CFI, TLI, SRMR, and PClose were assessed to check whether the provided data fit the suggested model.

As shown in Table 3, the obtained data resulted in acceptable to excellent goodness of fit indices. Accordingly, it can be reasonably concluded that the suggested model provided an acceptable fit with the given data.

**Table 3:** Goodness of Fit Indices.

**Criteria**		**Threshold**			**Evaluation**
		**Terrible**	**Acceptable**	**Excellent**	
CMIN	714.472				
df	366				
CMIN/df	1.952	> 5	> 3	> 1	Excellent
RMSEA	0.057	> 0.08	< 0.08	< 0.06	Excellent
CFI	0.929	< 0.9	> 0.9	> 0.95	Acceptable
TLI	0.921	< 0.9	> 0.9	> 0.95	Acceptable
SRMR	0.067	> 0.1	> 0.08	< 0.08	Excellent
PClose	0.062	< 0.01	< 0.05	> 0.05	Excellent

**Figure 2:**
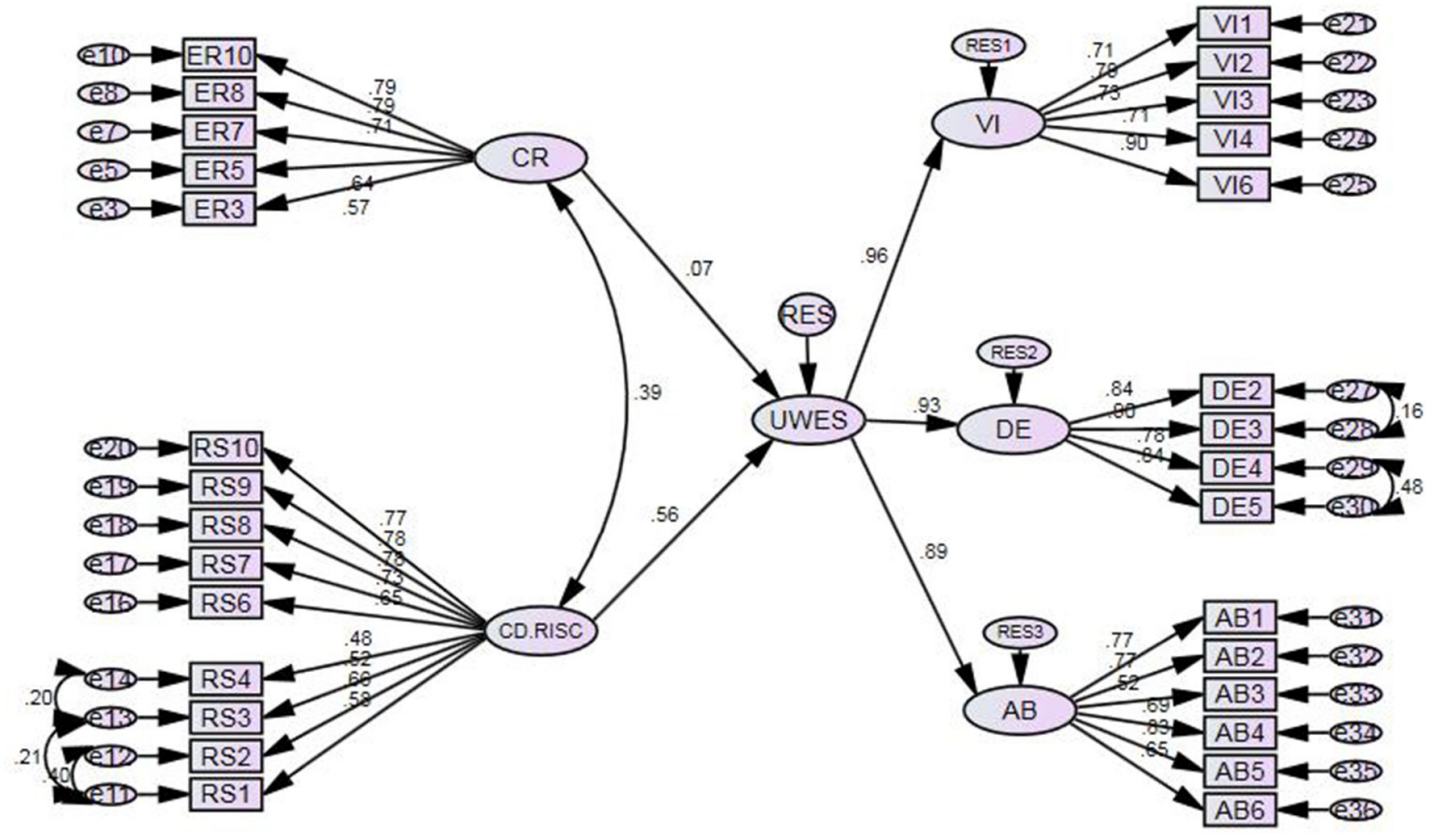
The structural equation model of the interrelationships among Chinese EFL teachers' resilience, cognitive reappraisal, and work engagement.

Figure 2 delineates the model of interrelationships among Chinese EFL teachers' resilience, cognitive reappraisal, and work engagement.

To identify how much of the variability in Chinese EFL teachers' work engagement could be accounted for by their resilience and cognitive reappraisal, the standardized estimates were measured. As Figure 2 depicts, resilience is found to be the significant antecedent (β = 0.558, *p* = 0.000 < 0.01) of Chinese EFL teachers' work engagement, whereas cognitive reappraisal failed to significantly predict their teaching engagement (β = 0.558, *p* = 0.000 < 0.01).

### The Qualitative Findings

**Figure 3:**
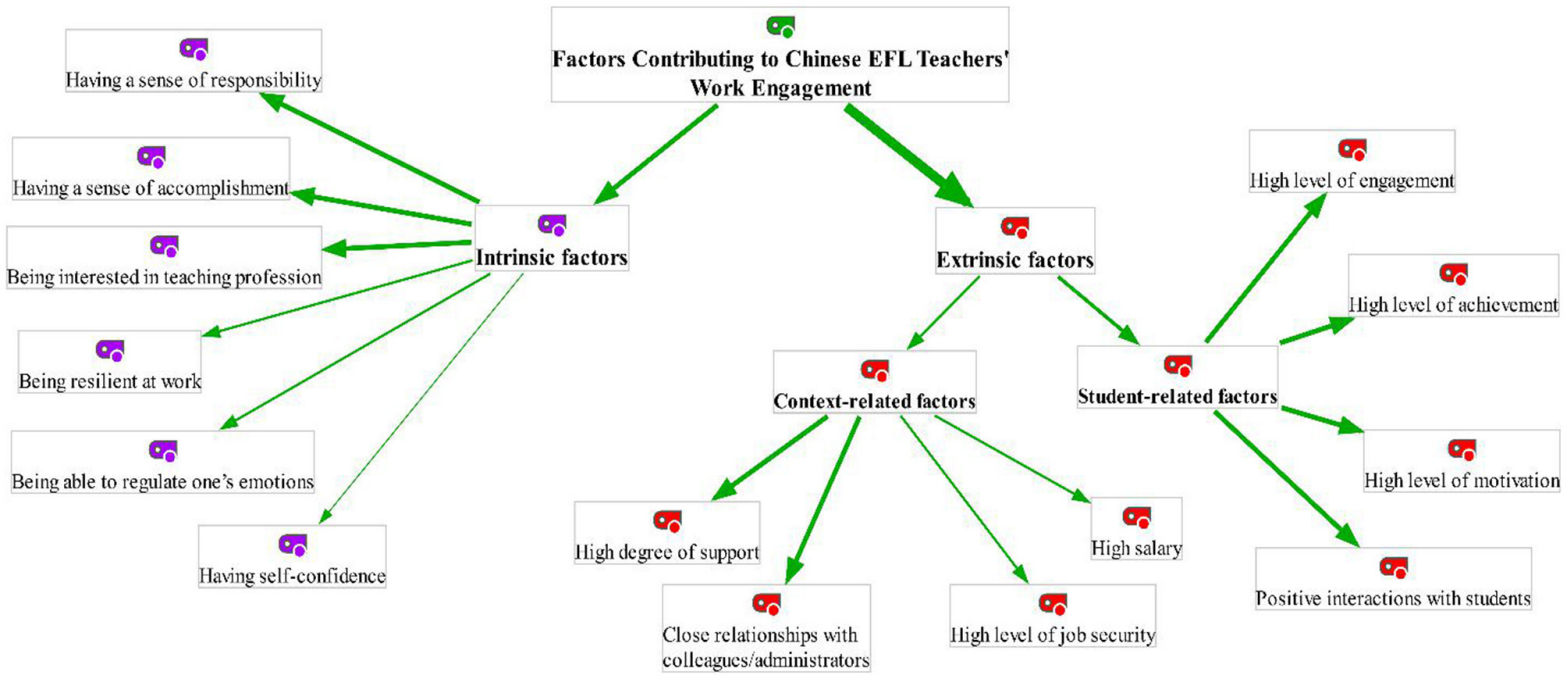
Factors contributing to Chinese EFL teachers' work engagement.

To thoroughly apprehend Chinese EFL teachers' perceptions of teaching engagement, some interview sessions were conducted. Employing MAXQDA software (version 2020), Chinese EFL teachers' responses to interview questions were grouped under two main themes, namely intrinsic factors (six sub-themes) and extrinsic factors (eight sub-themes) (Figure 3). The first group of themes was concerned with intrinsic factors contributing to teaching engagement. Based on the interviewees' responses, *having a sense of responsibility, having a sense of accomplishment*, and *being interested in the teaching profession* are the prime instances of intrinsic factors that can contribute to increased teaching engagement. These sub-theme can easily be identified from the following extracts:

P3: *I think the responsibility of being a teacher plays a very important role in my academic engagement*.
P7: *Sense of accomplishment can help me to perform better in my profession*.
P9: *I think being interested in what I'm doing can keep me motivated to work harder*.

Some participants referred to *being resilient at work, being able to regulate one's emotions*, and *having self-confidence* as other intrinsic antecedents of teaching engagement. Some statements highlighting these sub-themes are:

P1: *I think the ability to adapt to diverse environments is a most for being an engaged teacher*.
P11: *To me, those teachers who intend to be an engaged and effective teacher should be able to navigate their emotional experiences*.
P12: *Teachers with a high degree of self-confidence are more likely to engage in their profession*.

The second group of themes (i.e., extrinsic factors) revolved around *context-related factors* (four sub-themes) and *student-related factors* (four sub-themes). With regard to *context-related factors*, Chinese EFL teachers perceived that *high degree of support, close relationships with colleagues/administrators, high level of job security*, and *high salary* are critical predictors of their work engagement. To illustrate,

P2: *Support and trust are two important things we need at work and having these two features in our interactions with administrators and colleagues can dramatically increase our professional commitment*.
P4: *A close relationship with administrators and my colleagues can inspire me to work harder*.
P8: *I do believe that providing job security for teachers is what helps them stay engaged in their profession*.
P10: *I need money to support myself and my family. As such, high salary as a motivational factor can encourage me to strongly dedicate myself to my job*.

Finally, regarding the role of *student-related factors* in enhancing teaching engagement, four important sub-themes emerged from the interviewees' responses: *High level of engagement, high level of achievement, high level of motivation*, and *positive interactions with students*. The following extracts from interviewees' answers illustrate the aforementioned sub-themes respectively:

P1: *I think students' engagement can positively affect my level of engagement more than anything else*.
P5: *My students' high levels of achievement can inspire me to put more effort in transmitting knowledge*.
P6: *I think motivated students can encourage their instructors to become more active*.
P9: *Establishing rapport with students can make teachers more engaged in what they do*.

## Discussion

The primary purpose of the present inquiry was to assess the interrelationships between Chinese EFL teachers' emotion regulation (i.e., expressive suppression, cognitive reappraisal), resilience, and their work engagement. The results of correlational analyses revealed, first, a strong association between resilience and work engagement, and second, a moderate correlation between cognitive reappraisal and work engagement. Surprisingly, no significant correlation was found between expressive suppression and work engagement.

The association between teacher resilience and work engagement can be readily justified by the fact that those teachers who are able to navigate the difficulties and challenges of instruction receive a great deal of satisfaction from their profession. This, in turn, inspire teachers to become more engaged in their vocation (Mansfield et al., 2016; Polat and Iskender, 2018). In this regard, Fathi and Saeedian (2020) also stated that the capacity to adapt to adverse conditions encourages teachers to get more involved in instructional activities. The relationship between cognitive reappraisal and work engagement may also be explicated by the fact that those teachers who are capable of modifying, assessing, and regulating their positive and negative emotions are more likely to put effort into their profession (Sutton, 2004; Braun et al., 2020).

As an ancillary goal, the present study endeavored to examine the power of resilience and emotion regulation in predicting Chinese EFL teachers' work engagement. The results of SEM analysis revealed that Chinese EFL teachers' work engagement is predicted significantly and favorably by their resilience. This finding confirms the ideas of Tait (2008), who stated that teacher resilience as a valuable capacity can decrease instructors' sense of dissatisfaction and frustration, which in turn improves their professional commitment. The predictability of teachers' work engagement through their resilience is in congruence with Fathi and Saeedian's (Fathi and Saeedian, 2020) findings which indicated that EFL teachers' resilience can significantly contribute to their increased academic engagement. This result is also indirectly in agreement with those of Polat and Iskender (2018), who found a negative association between teacher resilience and burnout. In contrast to teacher resilience, Chinese EFL teachers' emotion regulation failed to significantly predict their work engagement. This finding contradicts the results of some published studies (Brackett et al., 2010; Chang, 2020; Fathi et al., 2021; Greenier et al., 2021; Mérida-López et al., 2021), which revealed that teachers' cognitive reappraisal and expressive suppression, two components of emotion regulation, could substantially predict their work engagement.

In order to fully understand Chinese EFL teachers' perceptions of work engagement, some qualitative data were also gathered through semi-structured interviews. The thematic analysis of participants' responses culminated in two main themes, namely intrinsic factors and extrinsic factors (Figure 1). As shown in Figure 1, most of the factors contributing to Chinese EFL teachers' work engagement were grouped under extrinsic factors. It means that the majority of interviewees perceived extrinsic factors (i.e., context-related factors, student-related factors) as important antecedents of their teaching engagement. To put it differently, most of the Chinese EFL teachers held their students, colleagues, and administrators responsible for their increased academic engagement. To them, different extrinsic factors such as *high degree of support, close relationships with colleagues/administrators*, and *students' high level of engagement, achievement, and motivation* can considerably enhance their teaching engagement. This finding is in agreement with those of Bakker and Demerouti (2008), who found job resources and student-related variables as the main antecedents of teachers' work engagement.

Besides, some Chinese EFL teachers perceived intrinsic factors as critical predictors of their work engagement. They assumed that their personal resources such as *sense of responsibility, sense of accomplishment, being resilient at work*, and *being able to regulate one's emotions* can assist them to get more involved in their profession. This finding resonates with the outcomes of some previous studies (Bakker and Demerouti, 2008; Hultell and Gustavsson, 2011; Yuan and Zhang, 2017; Mérida-López et al., 2020; Xie and Derakhshan, 2021) highlighting the power of teachers' personal resources in predicting their work engagement.

## Conclusion

To broaden the scope of research on ESL/EFL teachers' work engagement, the present study aimed to examine the role of emotion regulation and resilience as predictors of Chinese EFL teachers' work engagement. The results of correlational and SEM analyses lead to a major theme: Chinese EFL teachers' work engagement is predicted dramatically and positively by their resilience. To put it simply, Chinese EFL teachers' resilience can contribute to their increased academic engagement. Further, with regard to the findings of thematic analysis, it can reasonably be inferred that the antecedents of teachers' work engagement are not restricted to their personal resources. That is, some extrinsic factors such as job resources can also substantially affect teachers' academic engagement.

These findings can be illuminating and beneficial for administrators and teacher trainers. Given the significance of resilience in predicting teachers' work engagement, teacher trainers should instruct both pre- and in-service language teachers on how to cope with the difficulties and challenges of instruction. In fact, the trainees should be equipped with the ability to adapt themselves to adverse conditions. Moreover, due to the fact that extrinsic factors (e.g., close relationships with administrators, high salary, job security, etc.) can also positively predict teaching engagement, administrators are required to emotionally, cognitively, and financially support teachers. They are also expected to reassure teachers about the future of their profession.

Finally, the findings of the present inquiry are restricted by two important limitations. First, the present inquiry was conducted in China in an English as a foreign language country. Future studies need to be carried out in other EFL/ESL contexts to find any probable differences in the findings. Second, the effects of situational variables such as age, gender, teaching experience, and academic degree were not examined. Further investigations on this topic are recommended to measure the mediating effects of these variables on the association between teacher emotional regulation, resilience, and work engagement.

## Data Availability Statement

The original contributions presented in the study are included in the article/supplementary material, further inquiries can be directed to the corresponding author/s.

## Ethics Statement

Ethical review and approval was not required for the study on human participants in accordance with the local legislation and institutional requirements. The patients/participants provided their written informed consent to participate in this study.

## Author Contributions

The author confirms being the sole contributor of this work and has approved it for publication.

## Conflict of Interest

The author declares that the research was conducted in the absence of any commercial or financial relationships that could be construed as a potential conflict of interest.

## Publisher's Note

All claims expressed in this article are solely those of the authors and do not necessarily represent those of their affiliated organizations, or those of the publisher, the editors and the reviewers. Any product that may be evaluated in this article, or claim that may be made by its manufacturer, is not guaranteed or endorsed by the publisher.
